# Beyond borders: exploring the impact of augmented reality on intercultural competence and L2 learning motivation in EFL learners

**DOI:** 10.3389/fpsyg.2023.1234905

**Published:** 2023-10-04

**Authors:** Song Liu, Shengbing Gao, Xiaoyan Ji

**Affiliations:** ^1^School of Foreign Languages, Southeast University, Nanjing, China; ^2^School of Humanities and Tourism, Jiangsu Vocational College of Business, Nantong, China

**Keywords:** augmented reality, intercultural competence, L2 learning motivation, EFL learners, mixed methods study

## Abstract

**Introduction:**

This mixed-methods study investigates the impact of augmented reality (AR) on the development of intercultural competence and L2 (second language) learning motivation among Chinese English as a Foreign Language (EFL) learners. The research comprised forty-eight intermediate-level learners who were randomly assigned to either an experimental group, receiving AR-based language instruction, or a control group, receiving traditional instruction.

**Methods:**

Both groups underwent pre- and post-tests to assess their intercultural competence and L2 learning motivation. The experimental group engaged with an AR application, which exposed learners to a variety of cultural scenarios, customs, and norms.

**Results:**

The results indicate that the experimental group, exposed to AR-based instruction, demonstrated significantly higher levels of intercultural competence and L2 learning motivation in comparison to the control group.

**Discussion:**

Qualitative data analysis further elucidated that AR-based instruction enhanced learners’ engagement, motivation, and deepened their cultural understanding. This study highlights the potential of augmented reality as a powerful tool for fostering the development of intercultural competence and L2 learning motivation within the EFL context, suggesting promising opportunities for innovative pedagogical approaches in language education.

## 1. Introduction

Technology-enhanced learning has much more gained popularity due to the growth of information technology and mobile application (Ozcelik and Acarturk, 2011; Hwang and Wu, 2014; Hwang et al., 2015; Gambo et al., 2017; Teng et al., 2018; Lei et al., 2022). Traditional second language (L2) instruction methods might create challenges for both students and learners in today’s globalized world and they often fall short in providing authentic experiences that can enhance engagement and motivation (Wang and Vásquez, 2012; Fathi and Rahimi, 2022). As such, with the advancement of technology, new possibilities for language learning emerge, offering innovative and engaging approaches to address these challenges (Grgurović et al., 2013; Rahimi and Fathi, 2022). Augmented reality (AR), as a newly developing cutting-edge technology, has proven to be a beneficial tool for increasing electronic language learning and motivation (Mahadzir and Phung, 2013; Godwin-Jones, 2016; Li and Wong, 2021; Lin et al., 2022). Following Azuma (1997), AR is regarded as a three-dimensional technology that seamlessly blends both the virtual and real worlds in order to enhance learners’ understanding of the actual world with simulated items. As contextualized learning aids students in understanding and applying what they have learned to attain knowledge internalization, especially in the English as a foreign language (EFL) context (Chen and Li, 2010), the usage of AR in contextualized education is favorable for successful learning since AR combines virtual and real surroundings (Hsu, 2017; Chen et al., 2019; Reipschläger and Dachselt, 2019; Lee, 2022).

AR provides EFL students with cutting-edge audiovisual opportunities for learning and allows them to communicate with simulated information in real-world contexts which can lead to more immediate recall and improved comprehension of the learning material and motivation (Casella and Coelho, 2013; Zhao, 2018; Parmaxi and Demetriou, 2020). On the other hand, by adding technological data to the actual environment, AR presents fascinating novel possibilities that can improve the way learners see the outside world (Krüger et al., 2019; Zafar and Zachar, 2020). A number of studies have explored the role of AR in enhancing language learning performance in the English language contexts. For example, Richardson (2016) investigating the impact of AR game-based activities on EFL learners’ language learning, indicated that AR gamification improved the learners’ language learning performance. Sydorenko et al. (2019) also examining learners’ language learning via AR, found that AR could significantly contribute to the learners’ language learning, especially lexical resources.

More specifically, previous research has addressed the role of AR in enhancing English language learners’ intercultural competence, their language learning motivation, and their positive perceptions toward AR language learning environments. For instance, Matveev et al. (2021) investigating the impact of using AR in developing learners’ intercultural competence, indicated that the AR environment played a crucial role in improving the learners’ intercultural competence. Hadjistassou et al. (2021a,b) further found that the AR environment improved the learners’ intercultural competence. Kamarudin et al. (2021) investigating EFL students’ perceptions of virtual language learning applying an AR application, found that the AR application promoted the learners’ engagement in language learning activities. Yang and Mei (2018) also found learners’ positive perceptions toward the AR language learning environment.

Although the idea of ubiquitous learning (i.e., learning anywhere and anytime) has recently become widely recognized (Hwang and Tsai, 2011), the use of AR in actual educational settings has been constrained by a dearth of research and development of educational AR environments. The review of the literature revealed that AR played a substantial role in developing English language learners’ language learning (Yang and Mei, 2018; Sydorenko et al., 2019; Dalim et al., 2020; Li and Wong, 2021; Karacan and Polat, 2022), language learning engagement and motivation (Mahadzir and Phung, 2013; Richardson, 2016; Kamarudin et al., 2021; Wen, 2021), and intercultural competence (Liu et al., 2016; Hadjistassou et al., 2019; Matveev et al., 2021; Sabie et al., 2023). However, the impact of AR on learners’ learning motivation and intercultural competence needs further investigation, especially in the EFL context. To address this and get more insight into the literature, the present study examined the impact of AR on EFL learners’ learning motivation and intercultural competence. Qualitative data collection and analysis were further carried out exploring the learners’ perceptions of the impact of AR on their intercultural competence and language learning motivation. The following research questions, therefore, were postulated to address the purposes of the study:

1. Are there any significant differences between AR and non-AR classes in developing EFL learners’ intercultural competence and L2 learning motivation?
2. What are the perceptions of EFL learners toward the impact of AR on their intercultural competence and L2 learning motivation?

This study bridges this gap by examining the interplay between AR, intercultural competence, and L2 motivation—a triad that introduces unexplored dynamics and enriches the discourse on technology-driven language acquisition. In an increasingly interconnected world, the significance of intercultural competence for L2 learners is undeniable (Piątkowska, 2015; Avgousti, 2018; Liu et al., 2023). Our study recognizes the potential of technology-based approaches to amplify intercultural competence, especially for learners who face constraints in engaging with real-life cross-cultural interactions. Amidst this backdrop, our research acquires further distinction as it accentuates the importance of novel technologies like AR in enhancing intercultural competence. The unique two-fold purpose of AR—enhancing both communicative and intercultural competencies—might introduce a paradigm shift in pedagogical approaches.

In addition, central to our study’s distinctiveness is the innovative use of a custom-developed AR application. This application emerges as a conduit for cultural awareness and intercultural competence, crafting a holistic learning experience. Its distinctiveness lies in its dedication to infusing cultural richness into every aspect of learning, transcending the confines of language to cultivate adeptness in navigating cross-cultural interactions.

Also, our methodological fusion emerges as a distinguishing element. By employing a mixed methods approach, we unearth a deeper understanding of AR’s impact on intercultural competence and L2 learning motivation. Moreover, the demographics of our study participants—a group of undergraduate college students in China—add yet another layer of uniqueness to our research. This demographic, often characterized by intrinsic challenges in motivation towards English language communication and intercultural competence, defies initial expectations. Our findings, which highlight the engaging and motivating impact of AR on these participants, might reframe assumptions and underscore the dynamic nature of technology-enhanced language learning.

## 2. Literature review

### 2.1. Theoretical framework

The present study’s interactive cultural activities in both groups are grounded in Vygotsky’s (1984) social constructivism. Vygotsky’s perspective highlights that interactions between more capable individuals and learners gradually internalize and trigger higher levels of awareness. He posits that functions in cultural development manifest first on a social level and subsequently on an individual level—initially between individuals (inter-psychological) and later within individuals (intra-psychological) (Vygotsky, 1986, p. 57). Central to Vygotsky’s social constructivism is the concept of the Zone of Proximal Development (ZPD), wherein a gap exists between learners’ independent problem-solving ability and their potential growth when collaborating with more skilled peers. Through peer engagement and assistance, learners can bridge this gap and achieve their ZPD. Researchers contend that learners can alternate between the roles of more and less capable peers based on language learning tasks, leading to collaborative ZPD attainment across diverse skills (Kim, 2008). Sharing varying language abilities enables learners to collaborate, co-construct language skills, and achieve their ZPD.

In recent years, the rapid evolution of technology has introduced fresh possibilities for embedding Vygotsky’s principles within educational contexts (Fathi and Rahimi, 2022; Shadiev and Yu, 2022). While the core tenets of social constructivism remain timeless, contemporary educational research has explored how digital tools, portable devices, games, and adaptive learning experiences can enhance the effectiveness of social interactions and collaborative learning, aligning seamlessly with Vygotsky’s ideas (Lin and Lin, 2019; Liu et al., 2022). Digital platforms and technology-mediated environments offer learners unprecedented opportunities for collaborative activities, enabling them to fluidly transition between roles of knowledgeable peers and learners based on tasks (Tommerdahl et al., 2022). This versatility facilitates mutual support, collaborative problem-solving, and co-construction of knowledge, ultimately contributing to ZPD achievement.

Furthermore, modern educational technology, including Augmented Reality (AR), expands the horizons of collaborative learning (Yu et al., 2022). AR offers dynamic, immersive experiences that enable interactive engagement with cultural contexts and intercultural scenarios (Cai et al., 2022), aligning with Vygotsky’s emphasis on shared experiences and interactions. The integration of AR technology in the experimental group of this study provided learners with culturally enriched content and collaborative intercultural learning experiences, underscoring the symbiosis between technology and social constructivism. Similarly, learners in the control group collaborated with peers to explore their ZPD within traditional classroom settings, showcasing the continued relevance of Vygotsky’s principles across technology-enhanced and conventional learning environments.

In the evolving landscape of technology-enhanced learning, the fusion of Vygotsky’s social constructivism with contemporary digital tools accentuates the importance of collaborative interactions, co-constructed knowledge, and ZPD attainment through technology-mediated means (Fathi et al., 2023; Shortt et al., 2023). By embracing this synthesis, the present study contributes to the ongoing discourse on effective pedagogical approaches that leverage technology while remaining rooted in established educational theories.

### 2.2. Augmented reality

AR is conceptualized as “a situation in which a real-world context is dynamically overlaid with coherent location or context-sensitive virtual information” (Klopfer and Squire, 2008, p. 205). Following Carmigniani and Furht (2011), direct or indirect time perceptions of the outside world are enhanced by computer-generated digital data in AR. It is asserted that the idea of AR may now be seen broadly and is not restricted to any one sort of technology. For instance, mobile technologies, such as smartphones and tablets, may include AR games, which are thought to be beneficial for language learning purposes. Sannikov et al. (2015), similarly, argue that learners’ acquisition of skills and professional abilities is facilitated and expedited by the use of AR in mobile learning.

A substantial body of studies has examined the role of AR in second and foreign language contexts (Sydorenko et al., 2019; Parmaxi and Demetriou, 2020; Kamarudin et al., 2021; Karacan and Akoglu, 2021; Cai et al., 2022). Karacan and Polat (2022), for example, investigated the elements that influence pre-service English language teachers’ expectations to include AR in their subsequent language instruction. The teachers received instruction on how to incorporate AR activities and discussions into their language sessions. The results of a questionnaire showed that the perceived utility of AR was the most important predictor of the teachers’ willingness to utilize AR. The least important elements, however, were the favorable circumstances and ease of usage. Similarly, Li and Wong (2021) reviewing a number of studies about AR, indicated that a large number of studies have recently focused on the benefits of AR in educational settings. Li and Wong also revealed that most of the AR studies concentrated on the use of AR in developing learners’ speaking performance and vocabulary knowledge. They further recommend that along with AR, educators need to design appropriate curricular and learning materials in order to improve learners’ language proficiency.

In a similar vein, Kamarudin et al. (2021) examined how Saudi Arabian graduate and postgraduate EFL students behaved toward online learning via an AR application. They examined the social, personal, emotional, and cognitive variables that influenced the students’ electronic learning at higher education institutes. The findings revealed that interpersonal, social, and emotional aspects influenced the students’ behavioral goals and cognitive engagement with e-learning. Additionally, the findings showed that the moderating effect of cognitive involvement was used to explain how the students’ behavioral intentions toward electronic learning were impacted by personal, social, and emotional aspects. The findings suggested that EFL students need to be encouraged to learn by the AR application. Yang and Mei (2018) also explored learners’ perceptions of using AR for their language learning activities. Collecting the required data via semi-structured interviews, the findings indicated that the learners held positive perceptions of the application of AR for their language learning purposes.

Richardson (2016) examined the effects of AR-supported gamification in developing EFL learners’ language learning using the Aurasma mobile application. The learners accomplished a number of challenging language learning tasks via AR. The required data were gathered through observation and feedback on the learners’ language learning tasks accomplishments. The findings revealed that AR game-based activities enhanced the learners’ language learning and their engagement in language learning activities. Dalim et al. (2020) also investigated the influence of AR and speech recognition technologies on English language learners’ vocabulary knowledge gains and language learning engagement. An AR interface named TeachAR was developed for accomplishing the purposes of the study. Two experiments were applied to examine the effectiveness of combining AR and speech recognition in improving the learners’ vocabulary knowledge. The findings indicated that the learners in the experimental group outperformed their counterparts in the control group in terms of vocabulary knowledge and engagement. The findings also demonstrated that the combination of AR and speech recognition technologies helped the learners to accomplish certain language learning tasks more rapidly.

In a similar vein, Baabdullah et al. (2022) examined 500 Saudi Arabian undergraduate EFL students’ experience of using AR applications. The benefits of an AR environment on the personal, social, emotive, and cognitive dimensions of the EFL learners were examined. The findings demonstrated the strong influence of personal interactive, emotive, and cognitive benefits on the learners’ AR experiences. The outcomes also showed a substantial connection between the learners’ AR experience and their learning achievement. Ibáñez et al. (2020) also comparing the effectiveness of an AR learning environment and a web-based learning environment, indicated that the AR learning environment was more effective in developing the learners’ learning performance than the web-based learning environment. Furthermore, the learners who used AR demonstrated a higher level of motivation in the learning activities in comparison with the learners who did not benefit from AR.

Sydorenko et al. (2019) explored English language learners’ language learning via AR game-based activities. The data were gathered through video recordings of the learners’ interaction with AR using their mobile devices. Analyzing the data through the technique of language-related episodes, the findings indicated that in the AR environment, the learners mostly concentrated on lexical items. Following Vygotsky’s social constructivist theory of learning, the advice of more capable individuals and the immediate environment usually helped the learners understand the new lexical elements. Wen (2021) investigated English language learners’ motivation and engagement in an AR-supported environment. The results showed the learners’ high levels of motivation and engagement in AR-supported activities. The learners were more consistently involved with classroom activities that were made for their own settings as opposed to learning content knowledge created by experts.

Saleem et al. (2021) further explored 133 Pakistani university EFL students’ perspectives on online learning using AR during the COVID-19 pandemic. For data collection and analysis purposes, the study adopted structural equation modeling. Overall, the findings demonstrated that the EFL university students held positive perceptions toward using AR during the COVID-19 pandemic. Similarly, Li and Liu (2022) investigated college students’ perceptions of using AR for covering the content of their textbooks. The learners used mobile-based AR to browse the content of their textbooks more efficiently. The findings displayed that the students were strongly motivated and highly engaged in AR-supported activities, which could help them increase their learning performance. Moreover, Hsieh (2016) exploring both teachers’ and students’ perspectives on using AR materials via interviews, indicated that both teachers and students were willing and motivated to apply AR materials in their courses. The findings also revealed that using AR can positively influence both teachers’ and learners’ learning behavior.

The AR pop-up book is another option for helping students link the gap between the digital and physical worlds. By moving the book via the webcam and using detection—a two-dimensional pattern that carries information tied to the book page—students can use the AR pop-up book. Mahadzir and Phung (2013) created an AR pop-up book utilizing the ZooBurst technology and added a problem-solving strategy to aid with the motivation and language learning of English language students. The data were gathered through semi-structured interviews and observations of learners employing the AR pop-up book. The findings of their study showed that the learners’ motivation and language learning had improved.

Some studies have also focused on the role of AR in enhancing learners’ intercultural competence (Liu et al., 2016; Miranda Bojórquez et al., 2016; Hadjistassou et al., 2021a,b). For instance, Liu et al. (2016) examined the role of AR in improving English as a second language learners’ cultural awareness, communication skills, and language improvements. Gathering the data through video recordings, the findings revealed that the learners were highly engaged in both virtual and physical worlds and could successfully accomplish the required language learning tasks, which subsequently increased the learners’ cultural awareness, communication skills, and language learning. Matveev et al. (2021) examined the utilization of AR in forming learners’ multicultural competence. The Zome application was applied to engage the learners in an AR environment in order to enhance their multicultural competence which included the development of intercultural interaction and a new worldview. The findings revealed the positive role of AR in enhancing the formation of multicultural competence of the learners.

Sabie et al. (2023) investigated the role of AR in fostering intercultural exchanges among learners with different cultural backgrounds. An augmented reality application was utilized to link individuals with different cultures and help them interact with each other, so as to enhance their intercultural competence. The results indicated that the users were highly engaged in the AR environment because of the application’s narrative, visuals, and interactive features. Additionally, the users were more enthusiastic about exploring their own and other users’ cultural elements which boosted their confidence in connecting with individuals from other cultures. Hadjistassou et al. (2021a,b) explored how AR enhanced the intercultural competence of learners from two different academic institutions in Sweden and Cyprus. The findings demonstrated that the AR-based application provided game-based activities for the learners through which they were able to improve their intercultural competence. Similarly, Hadjistassou et al. (2019) examined the impact of AR on the intercultural competence of individuals with various cultural backgrounds. The learners were from two academic institutions in the United Kingdom and Cyprus. The findings indicated that AR could significantly contribute to both groups of learners’ intercultural competence.

### 2.3. AR-based and game-based learning technologies

Building upon the existing body of knowledge, a thorough exploration of the similarities and differences between AR-based learning and game-based learning technologies unveils interactions, shared traits, and distinct features that significantly shape the learning experience. AR dynamically merges real-world contexts with virtual information, resulting in a dynamic blend that enhances perception through digital data (Chen et al., 2019). On the flip side, game-based learning capitalizes on the intrinsic appeal of gaming to drive educational efforts (Kessler et al., 2022).

In the ever-evolving realm of mobile technologies, the convergence of AR and gamification takes center stage, exemplified by interactive platforms such as smartphones and tablets (Pellas et al., 2019). Within this dynamic landscape, AR-enhanced games thrive, offering promising avenues for nurturing language acquisition and cultural engagement (Chen, 2019). This cooperative partnership harnesses the inherent engagement of gaming to seamlessly intertwine interactive entertainment with language learning (Yu et al., 2022).

Comparatively, game-based learning, rooted in gamification principles, taps into the natural motivational allure of games to facilitate educational advancement. This approach adeptly incorporates educational content into the fabric of game narratives, fostering engagement, participation, and skill development (Hung et al., 2018). This synergy finds resonance in AR-enhanced language learning games, where real-world contexts intermingle with linguistic challenges, cultural experiences, and interactive scenarios (Cai et al., 2022). These fusion nurtures a comprehensive language proficiency and intercultural awareness.

Both AR-based learning and game-based learning harness interactivity and immersive experiences to cultivate learners’ motivation and dedication to learning (Chen et al., 2018). Rooted in the inherent joy and reward systems of gaming, both modalities fortify commitment and enthusiasm. Furthermore, both AR and game-based approaches facilitate experiential learning by immersing learners in genuine contexts, promoting practical language usage and cultural insights (Pellas et al., 2019). This immersion effectively bridges theoretical knowledge with real-world application. Additionally, interaction takes center stage in both approaches; AR-based learning involves dynamic engagement with digital overlays within real-world settings, while game-based learning emphasizes interactive challenges, narratives, and problem-solving tasks (Yu et al., 2022).

Nevertheless, AR surpasses its counterpart by enriching real-world contexts with virtual information, allowing for a deeper understanding of cultural intricacies and language application within genuine scenarios (Lin and Wang, 2023). Conversely, game-based learning frequently employs fictional scenarios to facilitate learning, providing learners with imaginative landscapes for language exploration. Narratives often drive game-based learning, embedding language and cultural learning within stories (Kessler et al., 2022). In contrast, AR-based learning immerses learners in authentic environments, bridging language skills with tangible real-world situations (Wen, 2021). Finally, AR seamlessly melds digital components with the physical world, facilitating instant connections between virtual tasks and the physical environment (Yang and Mei, 2018). Conversely, game-based learning flourishes within imaginative domains, imparting language learning within abstract contexts (Xu et al., 2020). This distinction underscores the varied ways in which these two approaches elevate the learning journey.

### 2.4. Intercultural competence in language learning

In the L2 domain, the learning process extends far beyond the mere acquisition of grammatical rules and vocabulary. In the increasingly interconnected world nowadays, achieving fluency in a foreign language is intimately intertwined with the ability to comprehend and navigate diverse cultural landscapes (O'Dowd, 2006; Wang, 2023). Beyond linguistic proficiency, the intricate interplay between language and culture transcends national boundaries, manifesting in the daily behaviors, interactions, and communication patterns of individuals (Kramsch, 2013; Byram and Golubeva, 2020). This interconnection finds its essence in the concept of intercultural competence—an essential capacity that enables effective and culturally apt engagement with individuals from a variety of cultural backgrounds (Byram, 1997, 2020).

Intercultural competence goes beyond the domain of linguistic prowess, delving into the realms of cultural sensitivity, awareness, and adaptability (Byram et al., 2013). Within the L2 context, it occupies a pivotal position in facilitating successful communication and cultivating profound interactions (O'Dowd, 2006; Wang, 2023). As the global landscape continues to evolve, individuals find themselves frequently engaged in interactions with people from diverse cultures—for academic, professional, or personal reasons. By nurturing intercultural competence, L2 learners acquire the skills to interpret and appreciate cultural subtleties, thereby enhancing the quality and effectiveness of their communicative endeavors (Tecedor and Vasseur, 2020).

The profound interrelationship between culture and language acquisition has been eloquently emphasized by Risager (2013), who asserts that language and culture are inherently intertwined. Language serves not only as a conduit for communication but also as a channel for conveying deeper meanings and inherited ways of life (Avgousti, 2018). An individual’s cultural background profoundly shapes their perceptions, interactions, and responses to diverse situations, with language serving as the medium through which these cultural inclinations find expression and mutual understanding (Avgousti, 2018; Byram, 2020).

Critical to effective intercultural communication, intercultural competence transcends mere linguistic mastery. Koester and Lustig (2010) stress that the L2 acquisition goes beyond the intricacies of language structure; it requires a nuanced understanding of the cultural dimensions that underpin communication. Intercultural competence empowers individuals to navigate cultural differences, discern subtle nuances, and communicate effectively across cultural boundaries. Byram (1997) introduces the concept of the “intercultural speaker,” an individual skilled in cross-cultural engagement. This competence encompasses dispositions, knowledge, and skills cultivated through exposure, reflection, and guided exploration (Byram et al., 2013; Byram and Golubeva, 2020). It involves an ongoing interplay between one’s own culture and the foreign culture, fostering a deeper appreciation for both. The intercultural speaker is an active participant, not a passive observer, who critically evaluates and adeptly adapts to varying cultural contexts (Byram, 2020).

To amplify intercultural competence, L2 education must transcend its traditional focus solely on linguistic proficiency. Byon (2007) underscores the significance of integrating cultural awareness into language teaching. Learners engage in interactive activities that simulate real-life cultural interactions, fostering enhanced cultural behaviors and attitudes (Shadiev and Yu, 2022). Educators play a pivotal role in guiding students through these immersive experiences (Liu et al., 2023).

In the assessment of intercultural competence, Portalla and Chen (2010) utilized the Intercultural Communication Effectiveness Measure, a comprehensive multidimensional construct. This framework delineates six distinct subscales—behavioral flexibility, interaction relaxation, interactant respect, message skills, identity maintenance, and interaction management—each encapsulating diverse facets of intercultural competence. Behavioral flexibility, a pivotal aspect, delves into participants’ adaptability within a panorama of intercultural interactions, showcasing their prowess in navigating diverse cultural contexts (Chen and Starosta, 2000). Interaction relaxation, another crucial element, is concerned with the emotional dimension of intercultural competence, exploring participants’ comfort levels during cross-cultural interactions. This offers insights into the emotional resonance experienced when engaging with individuals from diverse cultural backgrounds—an essential aspect of effective intercultural communication.

Interactant respect, a fundamental metric, assesses participants’ embrace of respect and open-mindedness when interacting with individuals from diverse cultural backgrounds. It deals with the demeanor and approach individuals adopt when engaging with others, underscoring the significance of mutual respect in fostering intercultural harmony (Chen and Starosta, 1997). Message skills, a dimension under scrutiny, examines participants’ ability to convey ideas and comprehend messages effectively within intercultural contexts (Chen, 2007).

Identity maintenance, another facet, investigates participants’ ability to uphold their cultural identity while engaging with cultures different from their own (Portalla and Chen, 2010). This dimension highlights the delicate balance between adaptation and preservation, revealing the extent to which individuals remain anchored to their cultural roots within the realm of cultural diversity (Wood, 2008). Lastly, interaction management, the final dimension, explores the art of effectively navigating intercultural interactions. This encompasses the spectrum from conflict resolution to collaborative prowess, offering insights into how individuals orchestrate effective communication and collaboration across cultural boundaries (Koester and Olebe, 1988).

Collectively, these dimensions, as elucidated by Portalla and Chen (2010), weave an intricate tapestry of intercultural competence. In their diversity, they mirror the multifaceted nature of effective intercultural communication and underscore the pivotal role such competence plays in fostering harmony, understanding, and successful collaboration within a diverse global landscape. In essence, intercultural communicative competence (ICC) encompasses a range of dimensions—intercultural communicative awareness, sensitivity, and effectiveness (ICE). ICE, closely aligned with the behavioral facet of ICC, revolves around the verbal and non-verbal communication behaviors crucial for intercultural collaboration (Chen and Starosta, 2007). This construct encapsulates the holistic essence of intercultural competence, anchoring it as a vital cornerstone in the realm of language learning.

## 3. Method

### 3.1. Participants

The current mixed methods study included 48 undergraduate students who were enrolled in a mandatory College English Band III course at a comprehensive interdisciplinary university situated in a suburban region of mainland China. The primary aim of the course was to enhance their proficiency in the English language. These individuals were categorized as intermediate level based on their English language scores derived from the annual national college entrance examinations in China. With an average age of 19.68 years (SD = 2.31), the participants were selected from two separate classes taught by an instructor who maintained independence from the researcher. In terms of prior experience with AR technologies, approximately 65% of the participants reported having some familiarity with AR applications, primarily from casual usage and exposure to mobile applications or games. Employing a quasi-experimental design, the participants were divided into either the experimental group (*n* = 25) or the control group (*n* = 23).

To ensure comparability between the groups, an independent-sample *t*-test was conducted employing scores from a recent college English language proficiency quiz completed by the participants. The findings revealed that students from both classes demonstrated a comparable level of proficiency in the English language, *t*(46) = −2.35, *p* = 0.152. Among the participants in the experimental group, eight students voluntarily agreed to partake in semi-structured interviews subsequent to the intervention. The selection of these participants was based on their willingness to engage in the interview process and their diverse backgrounds, including both prior AR experience and newcomers to the technology. It is noteworthy that student participation was entirely voluntary, and throughout the study, stringent measures were implemented to preserve the anonymity and privacy of the participants, adhering to ethical considerations and ensuring confidentiality.

### 3.2. Instruments

#### 3.2.1. Intercultural communicative competence

To assess participants’ intercultural competence in both groups, the Intercultural Communication Effectiveness Measure developed by Portalla and Chen (2010) was employed as a robust and established instrument. This measure comprised 20 items, each rated on a 5-point Likert scale, spanning from “strongly disagree” to “strongly agree.” The questionnaire encompassed six distinct subscales, namely behavioral flexibility, interaction relaxation, interactant respect, message skills, identity maintenance, and interaction management, which collectively capture various dimensions of intercultural competence.

The internal consistency of the entire scale was rigorously evaluated using the widely accepted Cronbach’s Alpha formula. The pre-test demonstrated a reliability coefficient of 0.86, indicating a high level of internal consistency, while the post-test exhibited a reliability coefficient of 0.82, signifying a satisfactory level of internal consistency for the scale. These findings underscore the reliability and stability of the instrument in measuring intercultural competence among the participants.

#### 3.2.2. Second language motivated behavior

To gauge participants’ motivation in learning English as a second language, this study employed the Motivated Behavior Scale developed by Taguchi et al. (2009), a widely recognized assessment tool in the field. The scale consisted of 10 self-report items, each specifically crafted to measure distinct facets of EFL learners’ motivated behavior. Participants were requested to indicate their level of agreement with each item on a five-point Likert scale, ranging from 1 (strongly disagree) to 5 (strongly agree).

One representative item from the scale was: “I am willing to invest substantial effort into mastering English.” To ensure the reliability of the scale, the internal consistency was examined using Cronbach’s alpha coefficient, yielding a reported reliability index of 0.88, indicating high internal consistency and stability of the instrument.

#### 3.2.3. Semi-structured interview

In order to gain a nuanced understanding of participants’ experiences, perceptions, and attitudes towards the AR Language Learning App and its influence on intercultural competence and L2 learning motivation, semi-structured interviews were employed. This qualitative research instrument facilitated an in-depth exploration of individual viewpoints, allowing participants to elaborate on their interactions with the app.

The semi-structured interviews adhered to a flexible interview guide, ensuring consistency while also permitting spontaneous and open-ended responses (see Appendix for details). These interviews were conducted individually with a selected subset of participants from the experimental group. A purposive sampling approach was employed to deliberately select participants who actively engaged with the app, representing a diverse range of experiences and perspectives. Eight participants willingly volunteered to take part in the interviews, thereby providing valuable insights from varied vantage points.

To create a comfortable and private environment conducive to open and honest dialog, the interviews were conducted in a relaxed setting. With the participants’ consent, the sessions were audio-recorded to ensure accurate data collection. The interviews lasted between 30 and 45 min, allowing ample time for participants to express their thoughts and reflect upon their experiences with the AR Language Learning App.

During the analysis phase, the audio recordings were meticulously transcribed verbatim, capturing both verbal and non-verbal cues. Thematic analysis was employed to identify prevalent patterns, recurring themes, and noteworthy excerpts related to intercultural competence and L2 learning motivation. The data derived from the semi-structured interviews served to complement and enrich the quantitative findings, fostering a more comprehensive understanding of participants’ perspectives and the overall impact of the AR Language Learning App.

#### 3.2.4. AR language learning app

In this study, we utilized a custom-developed AR Language Learning App specifically designed for the purposes of the research. The app was created by a team of experienced developers and instructional designers in collaboration with language teaching experts. It incorporated augmented reality technology to provide learners with an immersive and interactive language learning experience. This app was designed to deliver culturally rich content, interactive language exercises, quizzes, cultural challenges, and scavenger hunts to enhance language acquisition and intercultural competence. Upon launching the app, learners were greeted by an aesthetically pleasing dashboard, providing good access to a diverse range of features and functionalities. The application was thoughtfully designed to deliver a comprehensive and engaging learning experience, incorporating the following elements:

1. **Immersive Cultural Content:** The app curated an array of culturally enriched content, offering virtual tours of iconic cultural landmarks, interactive simulations of real-life scenarios, and multimedia presentations showcasing cultural traditions and practices.
2. **Interactive Language Exercises:** Learners could actively participate in a variety of interactive language exercises tailored to enhance their linguistic proficiency. These exercises encompassed vocabulary drills, grammar quizzes, and language comprehension activities, all carefully crafted to be both engaging and pedagogically effective.
3. **Quizzes and Assessments:** Beyond language skills, the application featured quizzes and assessments that encompassed intercultural knowledge evaluation. This comprehensive approach ensured a holistic assessment of learners’ progress, measuring both language competence and intercultural awareness.
4. **Cultural Challenges:** Promoting intercultural competence, the app presented learners with thought-provoking cultural challenges. These challenges encouraged learners to delve into the intricate cultural nuances of the language they were mastering, fostering a deeper understanding of intercultural communication dynamics.
5. **Scavenger Hunts:** Within the augmented reality environment, the app introduced captivating scavenger hunts, seamlessly merging language learning with real-world exploration. Learners were tasked with locating cultural artifacts and landmarks, thereby reinforcing language acquisition through practical, context-driven experiences.

### 3.3. Procedure

In this study, we divided the participants into two groups: the experimental group and the control group. The experimental group received the AR Language Learning App intervention, while the control group underwent traditional instruction without the use of AR technology.

Guided by the expertise of the same seasoned instructor, both groups embarked on their language learning paths with tailored approaches and activities attuned to their instructional methodologies. For the experimental group, the teacher introduced the AR Language Learning App to the participants and provided detailed instructions on how to use it. The app was designed with a user-friendly interface, allowing learners to easily navigate through its sections and activities. During the language classes, the teacher actively facilitated the learners’ engagement with the app, which offered a variety of culturally immersive situations and environments to explore. These included virtual shopping experiences, simulated dining scenarios, social gatherings, and simulated business interactions. Within these contexts, learners had the opportunity to interact with virtual characters representing individuals from different cultures. The teacher encouraged learners to have conversations with these virtual characters, promoting language practice and providing insights into cultural norms, customs, and communication styles.

To enhance language learning and intercultural competence, the app included interactive language exercises, quizzes, cultural challenges, and scavenger hunts. Learners were presented with specific language tasks within the app’s augmented reality environment. These tasks required them to label objects, construct sentences, respond to cultural questions, and identify cultural artifacts or landmarks in their physical surroundings. The activities were designed to actively engage learners in language acquisition and deepen their understanding of cultural diversity.

In the control group, the teacher employed established teaching techniques such as lectures, discussions, pair and group work, and exercises, similar to the experimental group. The instructional content focused on developing language skills in listening, speaking, reading, and writing, as well as grammar and vocabulary. To ensure parity with the experimental group, the teacher integrated cultural topics and contexts into the instruction. This involved discussing cultural practices, traditions, and customs during relevant language activities and exercises.

Throughout the control group sessions, the teacher facilitated interactive activities that aimed to reinforce language skills and knowledge while incorporating cultural aspects. These activities included communicative tasks, practice exercises, and drills, which were designed to improve language proficiency and provide opportunities for language practice within cultural themes. The teacher provided feedback on learners’ language performance through traditional means, such as verbal feedback, written corrections, and assessments.

It is important to note that the teacher played a crucial role in implementing the distinct instructional methods for each group while ensuring that cultural topics were integrated into the control group’s instruction. In the experimental group, the teacher focused on guiding learners’ interaction with the AR Language Learning App and promoting engagement with its culturally rich content. In the control group, the teacher utilized established teaching techniques, integrated cultural topics into language activities, and utilized traditional instructional materials to support language acquisition.

Evidently, the AR application’s content was purposefully curated to provide learners with dynamic opportunities for language acquisition and intercultural understanding. Through its immersive scenarios, interactive language exercises, and cultural challenges, the app aimed to promote active engagement, motivation, and a deeper connection with both the language and the rich tapestry of diverse cultural contexts. This comprehensive approach ensured that the content presented not only facilitated language learning but also enriched the learners’ understanding of cultural intricacies, contributing to their overall motivation and intercultural competence. The instructor’s role in implementing distinct methodologies for each group further ensured the continuity and comparability of the instructional strategies across both groups.

Overall, the unique content presented to both groups held the potential to significantly influence their motivation to participate in the learning activities. In the experimental group, the app’s captivating scenarios and interactive tasks offered a fresh, innovative learning avenue. Engaging with virtual characters and navigating augmented reality environments could have fostered a sense of excitement and curiosity, driving students’ intrinsic motivation to actively participate in language learning. The immersive experiences likely kindled a sense of adventure and discovery, potentially amplifying students’ enthusiasm to engage with the content and engage in language practice.

In the control group, the integration of cultural dimensions within traditional instruction also played a pivotal role in shaping motivation. The discussions on cultural practices and traditions could have enhanced students’ sense of connection to the language they were learning. By contextualizing language within cultural contexts, the students might have perceived the learning process as more meaningful and relevant, further fueling their motivation to participate.

In essence, the unique content presented to both groups had the potential to influence motivation through different pathways. The experimental group’s engagement with immersive augmented reality scenarios could have triggered curiosity and excitement, while the control group’s exposure to culturally woven traditional instruction could have evoked a deeper sense of purpose and connection. The interplay between content, instructional methods, and motivation forms a complex yet intriguing facet of this study’s exploration into enhanced language learning experiences.

### 3.4. Data analysis

A combination of quantitative and qualitative analysis methods was employed to evaluate the impact of AR technology on intercultural competence and L2 learning motivation. The quantitative data were summarized using descriptive statistics, offering insights into participants’ intercultural competence levels and L2 learning motivation. Paired samples *t*-tests were conducted to examine changes within each group before and after the intervention, providing an assessment of the effectiveness of the AR Language Learning App and traditional instruction. To compare intercultural competence and L2 learning motivation between the experimental and control groups, a one-way ANCOVA was performed, taking into account pre-test scores as covariates. The qualitative data obtained from semi-structured interviews were analyzed using content analysis, revealing valuable information about participants’ experiences and attitudes. By employing this comprehensive approach, a robust evaluation of the impact of AR technology on intercultural competence and L2 learning motivation was achieved.

## 4. Results

### 4.1. Quantitative results

Table 1 presents the descriptive statistics of the variables included in the study, namely the intercultural communicative competence (ICC) and L2 motivation. As seen in Table 1, the pre-test ICC mean score for the experimental group (*M* = 2.88, SD = 0.49) was slightly higher than the control group (*M* = 2.70, SD = 0.57). In the post-test, the experimental group showed an increase in the mean score (*M* = 3.76, SD = 0.85) compared to the pre-test. Similarly, the control group also exhibited an increase in the mean score (*M* = 3.15, SD = 0.69). Regarding L2 motivation, the experimental group had a mean score of 2.98 (SD = 0.35) on the pre-test, while the control group had a slightly higher mean score of 3.04 (SD = 0.40). For motivation post-test scores, the experimental group had a higher mean score of 3.69 (SD = 0.43) than the control group with a mean score of 3.30 (SD = 0.62).

**Table 1:** Descriptive statistics.

	Group	*N*	Mean	Std. deviation	Std. error mean
Pre. ICC	Experimental	25	2.8864	0.49866	0.09973
	Control	23	2.7087	0.57754	0.12043
Post. ICC	Experimental	25	3.7620	0.85138	0.17028
	Control	23	3.1500	0.69364	0.14463
Pre. Motivation	Experimental	25	2.9807	0.35214	0.07043
	Control	23	3.0477	0.40009	0.08343
Post. Motivation	Experimental	25	3.6901	0.43032	0.08606
	Control	23	3.3010	0.62620	0.13057

Table 2 presents the results of the paired samples *t*-test conducted to assess the differences in intercultural competence and motivation between the experimental and control groups before and after the intervention.

**Table 2:** Paired samples test for intercultural competence and motivation.

Group	Pair	*M*	SD	*t*	df	Sig. (2-tailed)	Cohen’s d
Experimental	Pre.ICC - Post.ICC	−0.87560	0.82740	−5.291	24	0.000	0.46
Experimental	Pre. Motivation - Post. Motivation	−0.70943	0.08992	−39.449	24	0.000	0.64
Control	Pre.ICC - Post.ICC	−0.44130	0.67498	−3.136	22	0.005	0.25
Control	Pre. Motivation - Post. Motivation	−0.25333	0.53337	−2.278	22	0.033	0.19

For the experimental group, the paired samples *t*-test revealed a significant increase in intercultural competence from pre-intervention (*M* = 2.88, SD = 0.49) to post-intervention (*M* = 3.76, SD = 0.85), *t*(24) = −5.29, *p* < 0.001. This change was associated with a moderate effect size, Cohen’s d = 0.46. Similarly, the experimental group demonstrated a significant increase in motivation scores from pre-intervention (*M* = 2.98, SD = 0.35) to post-intervention (*M* = 3.69, SD = 0.43), *t*(24) = −39.44, *p* < 0.001, with a large effect size, Cohen’s d = 0.64.

In the control group, the paired samples *t*-test indicated a significant increase in intercultural competence from pre-intervention (*M* = 2.70, SD = 0.57) to post-intervention (*M* = 3.15, SD = 0.69), *t*(22) = −3.13, *p* = 0.005. This change was associated with a moderate effect size, Cohen’s d = 0.25. The control group also exhibited a significant increase in motivation scores from pre-intervention (*M* = 3.04, SD = 0.40) to post-intervention (*M* = 3.30, SD = 0.62), *t*(22) = −2.27, *p* = 0.033, with a small to moderate effect size, Cohen’s d = 0.19.

These findings suggest that both the experimental and control groups experienced increases in intercultural competence and motivation following the intervention. However, the experimental group demonstrated larger mean differences in both intercultural competence and motivation compared to the control group. To explore the between-group differences, ANCOVA was used.

Table 3 displays the results of the ANCOVA run to examine the effects of the AR-based instruction on ICC. The results revealed a statistically significant impact of Group variable on the ICC scores [*F*(1, 45) = 5.783, *p* = 0.020, ηp^2^ = 0.114]. This ηp^2^ value suggests that approximately 11.4% of the variance in ICC scores can be attributed to the Group variable (i.e., independent variable), reflecting a moderate effect size. This finding underscores a notable difference in ICC scores between the experimental and control groups after accounting for pre-test ICC scores. Specifically, the experimental group demonstrated a more substantial increase in ICC compared to the control group.

**Table 3:** ANCOVA results for the ICC scores.

Source	Type III sum of squares	df	Mean square	*F*	Sig.	Partial eta squared
Pre.ICC	4.134	1	4.134	7.802	0.008	0.148
Group	3.065	1	3.065	5.783	0.020	0.114
Error	23.847	45	0.530			

Similarly, Table 4 presents the results of the ANCOVA conducted to assess the effects of the AR on the L2 motivation scores. The analysis showed a significant effect on the L2 motivation scores [*F*(1, 45) = 17.284, *p* < 0.001, ηp^2^ = 0.278]. The ηp^2^ value, approximately 27.8%, signifies a substantial effect size. This outcome highlights a significant difference in L2 motivation scores between the experimental and control groups after controlling for pre-test L2 motivation scores. The experimental group exhibited significantly higher L2 motivation scores compared to the control group, indicative of the strong positive impact of integrating AR technology in language learning environments.

**Table 4:** ANCOVA results for the L2 motivation scores.

Source	Type III sum of squares	df	Mean square	*F*	Sig.	Partial eta squared
Motivation1	6.619	1	6.619	46.163	0.000	0.506
Group	2.478	1	2.478	17.284	0.000	0.278
Error	6.452	45	0.143			

Overall, the results from the ANCOVA analyses support the hypothesis that the AR-based instruction had a more positive impact on both intercultural competence and L2 motivation compared to traditional instruction. The experimental group showed higher ICC and L2 motivation scores compared to the control group, indicating the potential benefits of incorporating AR technology in language learning settings.

### 4.2. Qualitative results

Additionally, the qualitative analysis focused on extracting themes and insights from the semi-structured interviews conducted with a subset of participants from the experimental group. Thematic analysis was used to identify patterns and commonalities in participants’ experiences, engagement, motivation, and cultural understanding facilitated by the AR Language Learning App. As revealed by the thematic analysis, participants consistently expressed a heightened level of engagement and motivation while using the AR Language Learning App. They conveyed enthusiasm about the interactive and immersive nature of the app, which made language learning more enjoyable and captivating. One participant, for instance, articulated, “I felt genuinely motivated to learn when I could see and interact with the virtual characters and environments. It made the whole process more exciting and fueled my curiosity.”

Also, the AR-based instruction effectively facilitated the development of cultural understanding among participants. Engaging in virtual conversations with the culturally diverse virtual characters provided learners with valuable insights into different cultural norms, customs, and communication styles. Participants acknowledged that this exposure allowed them to develop a deeper appreciation and understanding of various cultures. One participant shared, “Interacting with the virtual characters gave me a new perspective on cultural diversity. It taught me how to adapt my language and behavior in different cultural contexts, which significantly improved my intercultural competence.”

Some participants reported that the AR app enhanced their ability to apply language skills and cultural knowledge in real-world contexts. The incorporation of cultural challenges and scavenger hunts encouraged participants to explore their physical environment and identify cultural artifacts or landmarks. This aspect of the app helped participants bridge the gap between virtual experiences and the real world. As one participant mentioned, “The scavenger hunts made me more attentive to cultural elements in my surroundings. I started noticing nuances in everyday life that I had never paid attention to before. It made me feel more connected to the language and culture.” Furthermore, it was found the AR-based instruction provided participants with a personalized and adaptive learning experience. The app’s progress tracking system allowed learners to monitor their performance and track their learning progress. Students appreciated the instant feedback provided by the app, enabling them to identify areas for improvement and tailor their learning strategies accordingly. A participant, for example, emphasized, “The app’s feedback system helped me recognize my strengths and weaknesses in language learning. It motivated me to work on my weaker areas and celebrate my progress along the way.”

These emerged themes and sample excerpts from the semi-structured interviews highlight the positive impact of AR technology on EFL participants’ engagement, motivation, and cultural understanding. The qualitative findings align with the quantitative results, further reinforcing the idea that integrating augmented reality technology in language instruction significantly contributes to the development of intercultural competence and L2 learning motivation in EFL learners.

## 5. Discussion

This study examined the effects of using AR in enhancing EFL learners’ intercultural competence and language learning motivation by adopting a mixed-methods approach for data collection and analysis. Initially, the quantitative findings indicated that the AR-mediated class significantly improved the learners’ intercultural competence and outperformed its non-AR counterpart in that regard. The findings in this regard are in agreement with the findings of Hadjistassou et al. (2019), Matveev et al. (2021), and Sabie et al. (2023) who corroborated the positive effects of AR on learners’ intercultural competence. This augmentation in competence can be attributed, at least in part, to the novel nature of the AR environment. The immersive experience in the AR class motivated learners to engage in more vibrant communication with their virtual peers, fostering an environment conducive to improving intercultural competence. In essence, the virtual exposure within the AR class encouraged students to interact within a new realm, potentially yielding beneficial outcomes for their intercultural competence.

In line with Vygotsky’s (1984) social constructivism, the virtual learners in the AR class served as mediators for the other learners’ intercultural competence. In the beginning, the learners engaged in communicative intercultural activities with their virtual partners, which, as suggested by Vygotsky (1984), might have helped them in controlling both their own and their peers’ intercultural competence. This control entails navigating the intricacies of cross-cultural communication, effectively adapting and responding within diverse contexts. Moreover, the developmental process of learners unfolded in stages, transitioning from external regulation to self-regulation over their intercultural competence. This transition encompassed a shift from relying on external prompts, such as communicative activities facilitated by virtual peers, to independently engaging in intercultural endeavors. Those who mastered self-regulation displayed an advanced ability to function autonomously and successfully execute intercultural tasks without external aid. Ultimately, those who exhibited adept self-control demonstrated enhanced autonomy and efficacy in navigating and completing intercultural activities, fostering a sense of self-reliance.

The findings also underscored a significant disparity in the performance of AR-enabled learners compared to their non-AR counterparts. AR learners showcased a higher level of self-regulation in managing their intercultural competence, a result that suggests the role of AR in cultivating an environment conducive to developing learners’ self-regulatory skills. These skills empower learners to actively engage with intercultural content, adapt their responses, and independently undertake intercultural activities, collectively enhancing their overall intercultural competence.

According to Liu et al.’s (2016) findings, the results mentioned above may possibly be a result of the cultural understanding that the students received from their communicative intercultural encounters with their virtual peers. Due to the fact that the students and their virtual classmates had various cultural backgrounds and capabilities, they were able to exchange cultural information, which the students found to be interesting. The AR learners’ interest in learning novel cultural concepts in a virtual setting might have potentially enhanced their intercultural competence. Another factor contributing to the AR learners’ superior intercultural competency may be their successful internalization of cultural aspects, which, as mentioned above, was the direct result of the learners’ intercultural activities in the AR environment.

From a broader perspective, these findings align with contemporary educational research, which has explored the augmentation of social interactions and collaborative learning through digital tools, portable devices, games, and adaptive learning experiences (Lin and Lin, 2019). This perspective underscores the unprecedented opportunities for learners to participate in collaborative activities, fluidly transitioning between roles as knowledgeable peers and learners depending on the task (Tommerdahl et al., 2022). This fluidity fosters mutual support, collaborative problem-solving, and knowledge co-construction, ultimately contributing to the attainment of their ZPD. Augmented Reality, as a modern technology, further amplifies the horizons of collaborative learning (Yu et al., 2022) by providing dynamic and immersive experiences that facilitate interactive engagement with cultural contexts and intercultural scenarios (Cai et al., 2022).

The quantitative findings of this study also shed light on the enhancement of learners’ language learning motivation through the AR-supported class, surpassing its non-AR counterpart in this aspect. These results align with the research conducted by Dalim et al. (2020), Kamarudin et al. (2021), Mahadzir and Phung (2013), Richardson (2016), and Wen (2021), collectively demonstrating that AR-based classes significantly contribute to learners’ engagement and motivation in language learning. The AR learners, immersed in communicative intercultural activities within an augmented environment, encountered novel experiences that contributed to the enhancement of their intercultural competence, a factor they found motivating. Conversely, the AR learners were able to leverage various features of the augmented environment to elevate their motivation across different intercultural activities, thereby enhancing both their intercultural competence and motivation.

AR amalgamates advanced technology and captivating educational content, prompting a pivotal query: What serves as the true motivator for learners—is it AR’s innovative technology or its engaging content? Unraveling these influences is pivotal to comprehending the multifaceted impact of AR on learning. The allure of AR technology stirs curiosity and interest among learners (Yu et al., 2022). This innovative approach broadens the horizons of learning, nurturing active involvement that propels curiosity-driven exploration (Karacan and Akoglu, 2021). The captivating environment crafted by AR spurs learners to participate actively, a phenomenon substantiated by research demonstrating the motivating potential of emerging technologies (Chen, 2019; Ibáñez et al., 2020; Cai et al., 2022).

Delving into the educational content of AR, a crucial insight emerges: thoughtfully curated content lies at the heart of its impact. The AR application functions as a versatile platform encompassing a spectrum of intercultural encounters, language exercises, interactive scenarios, and tailored challenges. Prior research underscores this concept, underscoring the pivotal role of engaging content in fostering motivation (Li and Liu, 2022; Lin and Wang, 2023). However, at the heart of this issue lies the dynamic interplay between the innovative technology of AR and its educational content. As learners’ curiosity develops, they come to realize that their motivation is not solely ignited by novelty; rather, it is nurtured and enriched by the substantial and valuable educational material that AR offers. This symbiotic interaction creates a connection where engaging content enhances the allure of AR, resulting in an uninterrupted source of motivation. Research strongly emphasizes the critical significance of integrating technology with compelling content to maintain long-lasting enthusiasm (Pellas et al., 2019).

This discussion extends beyond the conventional juxtaposition of technology against content, advocating for a holistic viewpoint. While new technology sparks initial interest, absorbing content is the propelling force that nurtures and intensifies motivation over time. This harmonious synthesis positions AR as a realm where technology and content converge to shape learners’ objectives. This perspective encourages further investigation, prompting a more profound exploration of the instructional design implications.

Overall, within the domain of AR’s impact, the interplay between technology and content emerges as the pivotal catalyst propelling learners’ motivation. This fusion results in an enriched learning journey that resonates with learners, enriches intercultural competence, and fuels motivation for language acquisition. This thorough exploration contributes to the ongoing discourse on technology-infused learning, fostering a deeper understanding of the diverse dimensions that shape education in the digital age. Ultimately, the integration of AR seamlessly melds technology and content, propelling learners toward gratifying learning experiences.

The qualitative findings reveal the positive impact of the AR-based instruction on engagement, motivation, and cultural understanding. Thematic analysis of interviews uncovered key insights into participants’ experiences. They consistently reported higher engagement and motivation, finding the interactive and immersive nature of the app enjoyable. This suggests that augmented reality technology effectively enhances motivation and active participation in language learning (Erbas and Demirer, 2019; Ibáñez et al., 2020).

Moreover, the AR-based instruction facilitated cultural understanding by allowing learners to engage in virtual conversations with diverse characters. This exposure provided valuable insights into cultural norms, customs, and communication styles, fostering appreciation and improving intercultural competence. The app also encouraged the application of language skills and cultural knowledge in real-world contexts through cultural challenges and scavenger hunts. By bridging the gap between virtual and real experiences, language learning became more meaningful and relevant (Liu et al., 2023; Parmaxi, 2023). Participants highly valued the personalized and adaptive learning experience offered by the app. The progress tracking system and instant feedback empowered learners to monitor their performance, identify areas for improvement, and tailor their strategies. This individualized approach enhanced self-awareness and motivated learners (Xie et al., 2019).

The qualitative findings complement the quantitative results, offering a comprehensive understanding of AR technology’s effects on intercultural competence and L2 learning motivation. Integrating augmented reality technology in language instruction shows promise in creating engaging, immersive experiences, fostering cultural understanding, and promoting learner autonomy. It is recommended that establishing an AR environment can involve students in a pleasant digital setting where they can communicate with their peers with greater efficiency, which can effectively boost their intercultural competence. The current study found that the EFL students were actively participating in user-friendly intercultural activities with their peers, which could effectively contribute to their intercultural competence and motivation. These communicative intercultural activities are believed to help students improve their capacity for self-regulating their intercultural competence. Vygotsky (1984) asserted that students with different skills and capabilities can help other students achieve their highest degree of performance. By fostering positive perceptions of the learners’ cultures and abilities, the AR class helped the learners in the current study engage in communicative intercultural tasks more effectively. Due to the diversity of cultural backgrounds among the learners who took part in the present research, the learners could greatly increase their peers’ intercultural competence and motivation.

## 6. Conclusion and implication

The goal of the current study was to examine an AR language learning environment that was created to assist EFL students in developing their intercultural competence and learning motivation. In line with Vygotsky’s social constructivism, the results showed that the AR class was more effective than the non-AR class in enhancing the intercultural competence and learning motivation of EFL learners. The findings were due to the innovative and engaging environment which was augmented by some virtual features. The learners also confirmed that they held positive perceptions toward language learning and intercultural activities experienced in the AR environment.

The findings pointed to a number of fruitful educational implications, especially for the EFL context. The AR class is suggested to be applied in interactive EFL intercultural courses since it is in line with modern ideas of student-centeredness and substantially enhances EFL students’ intercultural competence and language learning motivation. In order to improve the intercultural competence and motivation of EFL students, EFL educators, teachers, and students are encouraged to utilize an AR environment for their communicative intercultural activities. To provide EFL students with engaging communicative intercultural activities with other students, EFL educators and teachers might set up an AR class. This makes it possible for EFL students to participate in more communicative intercultural tasks that can more successfully increase their intercultural competence and language learning motivation. By taking an AR class made particularly for communicative intercultural activities, EFL students could benefit more from peer intercultural mediation. They can receive peer and AR mediation on intercultural issues and enhance their intercultural competence accordingly. Additionally, the more engaging and interesting communicative intercultural activities in the AR environment can boost EFL students’ intercultural competence and enthusiasm for language learning.

Although this study contributes valuable insights into the impact of AR on intercultural competence and L2 learning motivation among Chinese EFL learners, several limitations should be acknowledged. First, the participant pool consisted of undergraduate college students enrolled in a mandatory English course in China. This specific demographic may not fully represent the diversity of English language learners across various age groups and proficiency levels. The findings may be more applicable to similar contexts and may not be generalizable to other EFL learner populations, such as adult learners or those in different educational settings.

Second, the study employed a mixed methods approach, which allowed for a comprehensive exploration of the research questions. However, the scope of the qualitative analysis was limited to participants’ perceptions and experiences. A more in-depth qualitative exploration, potentially incorporating interviews and focus group discussions, could provide richer insights into the findings. Third, the duration of the study was relatively short, encompassing a single semester. Longer-term investigations could offer a deeper understanding of the sustainability of the observed effects over extended periods of time. Additionally, the study did not explore the potential influence of prior experience with AR technology on participants’ responses to AR-based language instruction. Future research could investigate whether prior familiarity with AR impacts learners’ engagement and motivation.

Fourth, while the custom-developed AR Language Learning App enriched the learning experience through its cultural content, the effectiveness of individual app features and modules was not isolated in this study. Further research could delve into the specific impact of different components of the app on learners’ intercultural competence and motivation. Finally, this study focused on the immediate impact of AR on intercultural competence and L2 learning motivation. Long-term effects and transfer of skills to real-life intercultural interactions were not within the scope of this investigation. A longitudinal study that traces participants’ experiences and behaviors beyond the instructional period could offer deeper insights into the sustained impact of AR-enhanced learning.

## Data availability statement

The raw data supporting the conclusions of this article will be made available by the authors, without undue reservation. Requests to access these datasets should be directed to SL: dndxjyjy@163.com.

## Ethics statement

The studies involving humans were approved by School of Foreign Languages, Southeast University, Jiangning District 211189, Nanjing, China. The studies were conducted in accordance with the local legislation and institutional requirements. The participants provided their written informed consent to participate in this study.

## Author contributions

All authors listed have made a substantial, direct, and intellectual contribution to the work and approved it for publication.

## Appendix

**Interview Questions**

1. How did the AR Language Learning App impact your overall language learning experience? Can you describe any specific instances where the app helped you overcome challenges or enhance your understanding?
2. What aspects of the AR Language Learning App did you find most valuable in terms of increasing your engagement and motivation to learn?
3. In what ways did the app contribute to your development of intercultural competence?
4. How did engaging in virtual conversations with culturally diverse virtual characters in the app enhance your cultural understanding and language skills? Can you discuss any specific insights or perspectives you gained through these interactions?
5. Looking back on your experience with the app, how do you believe it compared to other language learning methods or tools you have used? What advantages did the app offer that may have been lacking in other approaches?
